# Epigenetic Compound Library Screen Identifies Ibrutinib as an Inhibitor of Ovarian Clear Cell Carcinoma Viability

**DOI:** 10.1002/cam4.71795

**Published:** 2026-04-08

**Authors:** Yue Ma, Kristie‐Ann Dickson, Farhana A. Sarker, Amani Alghalayini, Natisha R. Field, Tao Xie, Tali S. Skipper, Anastasia Karafotias, Sarina Briscas, Christine Yee, Caroline E. Ford, Nikola A. Bowden, Nham Tran, Deborah J. Marsh

**Affiliations:** ^1^ Translational Oncology Group, School of Life Sciences, Faculty of Science University of Technology Sydney Ultimo New South Wales Australia; ^2^ Gynaecological Cancer Research Group, School of Clinical Medicine, Faculty of Medicine and Health University of New South Wales Sydney New South Wales Australia; ^3^ Hunter Medical Research Institute, New Lambton Heights New South Wales Australia; ^4^ School of Medicine and Public Health University of Newcastle Newcastle New South Wales Australia; ^5^ School of Biomedical Engineering, Faculty of Engineering and IT University of Technology Sydney Ultimo New South Wales Australia

**Keywords:** Bruton's tyrosine kinase, drug repurposing, ibrutinib, ovarian clear cell carcinoma

## Abstract

**Background:**

Ovarian clear cell carcinoma (OCCC) is an endometriosis‐associated ovarian cancer subtype. Somatic mutations in OCCC are reported in *ARID1A*, *PIK3CA*, and the *TERT* promoter (*TERTp*), as well as less commonly in *KRAS* and *TP53* among other genes. OCCC is typically resistant to standard‐of‐care chemotherapy, especially after relapse. While recent studies have seen favourable responses to immunotherapy, patients with OCCC face limited therapeutic options.

**Methods:**

With the objective of discovering new drug treatments for OCCC, we screened OCCC (RMG‐1, JHOC‐5, OV207, OVISE, OVMANA, OVTOKO, and TOV‐21G) and non‐OCCC cell lines with a commercially available epigenetic drug compound library at two concentrations. Based on specified selection criteria, drugs were sought that preferentially inhibited viability of OCCC versus non‐OCCC cells, with subsequent validation in 2D and 3D bioprinted models and exploration of a relevant signalling pathway.

**Results:**

Taken together, OCCC cell lines were more sensitive to the Bruton's Tyrosine Kinase (BTK) inhibitor ibrutinib than non‐OCCC cells, with some variation in response observed between cell lines in 2D and 3D bioprinted cultures. Furthermore, ibrutinib inhibited PI3K/AKT/mTOR cell survival signalling in some but not all OCCC cell lines, suggesting that this drug functions on additional pathways.

**Conclusions:**

Ibrutinib is used clinically to treat specific B cell disorders; however, it is not currently approved to treat solid tumours. Data presented in OCCC cell lines complements clinical observations of a therapeutic response to ibrutinib in low‐grade serous ovarian cancer. Ibrutinib demonstrates potential for the treatment of certain rare subtypes of ovarian cancer and should be further investigated.

## 1 Introduction

Ovarian clear cell carcinoma (OCCC) is a histological subtype of ovarian cancer with distinct clinical behaviour, morphological and molecular features relative to other ovarian cancer subtypes. OCCC can present as a solid, mixed solid and cystic, or mainly cystic mass [1]. The clear appearance of OCCC cells is attributed to accumulated cytoplasmic glycogen [2]. While OCCC is one of the rarer ovarian cancer subtypes in the USA [3, 4], in Taiwan and Japan it has been reported to constitute 20% and almost 27% of all ovarian cancer cases, respectively [5, 6]. While the frequency of OCCC as a percentage of overall ovarian cancers varies around the world, some studies suggest that ethnicity, rather than simply geographical location, plays a role in these differences [7, 8].

OCCC is recognised as an endometriosis‐associated cancer, and while it is important to state that the vast majority of women with endometriosis will never develop ovarian cancer, a recent registry‐based study reports that there is an elevated risk [9, 10]. In some cases, endometriotic lesions may in fact be precursors to the development of OCCC [11]. Of note, studies have also reported a higher frequency of endometriosis in Asian women (reviewed in [12]), providing a possible explanation for the higher proportion of OCCC in some Asian countries. Further studies are required to confirm these reports and observations.

While OCCC is typically diagnosed at a younger age and earlier stage than the more common subtype high‐grade serous ovarian cancer (HGSOC), this cancer has been shown to have shorter times to progression and a worse prognosis [8, 13, 14, 15, 16]. To date, standard of care for OCCC has been similar to HGSOC, specifically cytoreductive surgery and a combination of platinum‐based and paclitaxel chemotherapy [14]. However, only 11%–27% of women with OCCC respond to this treatment, with just 1%–2% still responsive at relapse [17, 18, 19]. Despite generally being considered as a malignancy with low mutational burden, recent studies of the potential of immune checkpoint blockade to treat OCCC, including with the anti‐PD‐1 antibody pembrolizumab, have demonstrated promising results [20, 21, 22, 23, 24, 25, 26].

OCCC have a distinct molecular profile relative to other ovarian cancer subtypes, with gene mutations frequently observed in *ARID1A*, *PIK3CA* and the *TERT* promoter (*TERTp*); as well as less frequently in genes including *KRAS*, *TP53*, and others [27, 28, 29, 30]. The high frequency of *ARID1A* mutation in OCCC, reported to be between 41.5%–67% of OCCC (reviewed in [30]), implicates defective chromatin modelling in this ovarian cancer subtype. Mutations in the oncogene *PIK3CA* have been shown to occur frequently with *ARID1A* mutation in OCCC [31, 32]. Numerous clinical trials are attempting to exploit synthetic lethal interactions with *ARID1A* mutations; however, none to date have resulted in an approved therapy for OCCC (reviewed in [23]). Here, we report the use of a commercially available epigenetic compound library to screen both OCCC and non‐OCCC cell lines to identify new opportunities for the treatment of OCCC.

## 2 Materials and Methods

### 2.1 Cell Lines

Seven OCCC cell lines (RMG‐1, JHOC‐5, OV207, OVISE, OVMANA, OVTOKO, and TOV‐21G) and seven non‐OCCC cell lines (A2780.b1, a clonal cell line from the A2780 parental endometrioid ovarian cancer (EnOC) cell line [33]), COV434, small‐cell carcinoma of the ovary, hypercalcaemic type (SCCOHT) cells, and high‐grade serous ovarian cancer (HGSOC) cell lines CaOV‐3, COV362, Kuramochi, OVCAR‐3, and OVCAR‐8 were used in this study.

Cell lines were sourced as follows: Kuramochi [cat. #JCRB0098], OVISE [cat. #JCRB1043], OVMANA [cat. #JCRB1045], OVTOKO [cat. #JCRB1048] and RMG‐1 [cat. #JCRB0172] (JCRB Japanese Collection of Research Bioresources), Osaka, Japan, distributed through CellBank Australia, NSW, Australia); COV434 [cat. #07071909] (European Collection of Authenticated Cell Cultures (ECACC), distributed through CellBank Australia); and OVCAR‐3 [cat. #HTB‐161, American Type Culture Collection (ATCC), VA, USA]. CaOV‐3 (gifted by Professor Anna Defazio, Westmead Institute for Medical Research, NSW, Australia); COV362 (gifted by Professor Nikola Bowden, Hunter Medical Research Institute, NSW, Australia); JHOC‐5 and OVCAR‐8 (gifted by Professor David Bowtell, Peter MacCallum Cancer Centre, VIC, Australia); OV207 (gifted by Dr. Kimberly Kalli and Professor Cheryl Conover, Mayo Clinic, MN, USA); and TOV‐21G (gifted by Professor Caroline Ford, University of NSW, NSW, Australia). The original A2780 cell line was a gift from Ms. R. Harvey (Bill Walsh Cancer Laboratory, Kolling Institute of Medical Research, Australia). All cell lines were cultured in a humidified incubator containing 5% CO_2_ at 37°C in the following media: A2780.b1, CaOV‐3, JHOC‐5, Kuramochi, OV207, OVCAR‐3, OVCAR‐8, OVISE, OVMANA, OVTOKO and RMG‐1 in RPMI 1640 (cat. #42402016, ThermoFisher Scientific, VIC, Australia) plus 10% v/v foetal bovine serum (FBS; cat. #FBSAU, AusGeneX, Molendinar, QLD, Australia); COV362, COV434 and TOV‐21G in DMEM (cat. #11965084, ThermoFisher Scientific) plus 10% FBS v/v. All cell lines underwent short tandem repeat (STR) profiling for authentication using the *GenePrint* 10 System (cat. #B9510, WI, USA) by the Australian Genome Research Facility (AGRF, VIC, Australia) and were shown to match previously reported analyses. Cell lines routinely tested negative for mycoplasma by the MycoAltert Mycoplasma Detection Kit (cat. #LT07‐318, LONZA, MD, USA). Of the OCCC cell lines, only RMG‐1 is wild‐type (WT) for both *ARID1A* and *ARID1B* . *ARID1A* mutations in OV207, OVISE, OVMANA, OVTOKO and TOV‐21G have been previously reported [34, 35]. OVISE and TOV‐21G harbour both *ARID1A* and *ARID1B* mutations. Cell line mutations are reported in the Cancer Dependency Map (#DepMap ID: ACH‐000324, DepMap Public 24Q4 (last accessed September 23, 2025). DepMap also reports an *ARID1A* mutation in JHOC‐5 that had previously been reported as an *ARID1A* WT cell line.

### 2.2 Epigenetic Drug Screening Library and Validation

The Tocriscreen Epigenetics Library 3.0 (cat. #7578, Tocris Bioscience, Bristol, UK), consisting of 160 epigenetic compounds, was used in a hypothesis generating screen to investigate seven OCCC cell line models (RMG‐1, JHOC‐5, OV207, OVTOKO, OVMANA, TOV‐21G and OVISE) and three non‐OCCC cell line models (A2780.b1, COV434 and OVCAR‐8). In brief, cells were seeded into 96‐well plates at the following seeding densities: A2780.b1 (5000), COV434 (25000), JHOC‐5 (2500), OV207 (2000), OVCAR‐8 (2500), OVISE (4500), OVMANA (4500), OVTOKO (2500), RMG‐1 (6000), and TOV‐21G (5000). Cells were treated in triplicate with either 5 μM or 0.5 μM of each compound in the drug library in a single experiment as is standard for a primary screen [36]. After 72 h, cell viability was assessed using the CellTiter 96 Aqueous One Solution Cell Proliferation Assay (cat. #G3581, Promega Corporation). Absorbance at 490 nm was measured using a Tecan Infinite 200 PRO microplate reader (Tecan, VIC, Australia). Data was then normalised to dimethyl sulfoxide (DMSO) vehicle‐treated cells. Post library screening, ibrutinib was purchased separately [cat. #HY‐10997, MedChemExpress, NJ, USA (distributed by Focus Bioscience)], with stock solutions utilised for subsequent experiments.

To validate the efficacy of ibrutinib, cell viability was measured after treatment for 72 h with either DMSO vehicle control or a 10‐point dilution series of ibrutinib from 50 to 0.004 μM (1:1.5‐fold from 50 to 14.8 μM, followed by 4‐fold dilutions from 14.8 to 0.004 μM) in cell media. This concentration range included the reported maximum plasma concentration (plasma C_max_) [37, 38, 39]. Cell viability, calculated as the average of triplicate values minus background readings (wells containing media only), was expressed as a percentage relative to DMSO vehicle control. Dose response curves were computed in GraphPad Prism v10.4.1 (GraphPad Software, MA, USA).

Area‐under‐the‐curve (AUC) analysis was also conducted in GraphPad Prism v10.4.1 using default parameters. Briefly, the software utilises the trapezoid rule to approximate AUC values, where straight lines were repeatedly drawn between adjacent data points and the areas under each segment summed across the curve. Area of each segment was calculated using the formula:

$$∆x\times \left(\frac{y_{1}-y_{2}}{2}\right)$$

AUC values were computed for each biological replicate (the mean of 3 technical replicates) and statistical comparisons were subsequently performed within GraphPad Prism software.

### 2.3 Western Blotting and Ibrutinib Treatment

Protein was extracted and western blotting was carried out as previously described [33]. Cells were treated with 5, 10 or 20 μM ibrutinib or DMSO vehicle control for 48 h prior to protein extraction. In brief, 10 μg of each sample was separated on a 4%–12% Bis–Tris gel (cat. #NP0336BOX, Life Technologies, NSW, Australia) and subsequently transferred to a nitrocellulose membrane (cat. #926–31,092, LI‐COR Bioscience, NE, USA). Membranes were blocked with Tris‐buffered saline (TBS) Intercept Blocking Buffer (cat. #927–60,001, LI‐COR Bioscience) and incubated overnight at 4°C with the following primary antibodies from Cell Signalling Technology (Danvers, USA): Akt (cat. #9272), Phospho‐Akt (Ser473) (cat. #9271); mTOR (cat. #2972); Phospho‐mTOR (Ser2448) (cat. #2971) and GAPDH (D14C10) (cat. #2118). Membranes were probed with the near infrared (NIR) fluorescent secondary antibody IRDye 800CW Donkey anti‐rabbit IgG (cat. #926–32,213, LI‐COR Bioscience), and imaged on the Odyssey CLx imaging system (LI‐COR Bioscience). Quantitation was undertaken using ImageStudio software version 5.2 (LI‐COR Bioscience).

### 2.4 3D Bioprinting and Ibrutinib Treatment

The RASTRUM 3D bioprinting platform (Inventia Life Science, NSW, Australia) was employed to generate 3D cell models utilising commercially purchased polyethylene glycol (PEG)‐based ‘bioinks’ and crosslinking activators that polymerise to form hydrogel matrices. Hydrogels were of an elastic modulus of 3.0 kPa and were biofunctionalised with adhesion peptide motif RGD (Arg‐Gly‐Asp) (*Px03.31P*, Inventia Life Science). Cells were encapsulated into biofunctionalised hydrogel as a ‘large plug’ model and deposited onto bases of inert hydrogel in 96‐well, tissue culture‐treated, low evaporation plates (cat. #CLS3595, Corning, NY, USA). Printing densities utilised for cell lines were: 5.56 × 10^6^ cells/mL for OVMANA, and 6.25 × 10^6^ cells/mL for JHOC‐5, OV207, OVISE, OVTOKO, RMG‐1, and TOV‐21G. Cell densities were optimised depending on cell size and growth rate, allowing for 3D structures to be formed 96 h post‐printing, permitting the addition of ibrutinib or DMSO vehicle control at this time point.

Bioprinted cell models were treated for 72 h with either DMSO vehicle control or a 10‐point dilution series of ibrutinib, as performed for 2D cell viability assays. Cell viability was assessed using CellTiter‐Glo‐3D bioluminescent reagent (cat. #G9681, Promega Corporation) as per the manufacturer's instructions, and luminescence measured using a Tecan Infinite 200 PRO microplate reader (Tecan). Cell viability, computed as the average of duplicate values, minus background readings (wells containing inert base and media only), was expressed as a percentage relative to DMSO vehicle control.

### 2.5 Live/Dead Imaging of Ibrutinib Treated OCCC Cells

Utilising the RASTRUM ‘imaging model’ format, OVMANA and RMG‐1 cells were bioprinted into black, clear‐bottomed 96‐well plates (cat. #6055302, Revvity, MA, USA). Cells were treated with ibrutinib at doses of 0.058 μM, 0.231 μM, 22.2 μM and 50 μM, or DMSO vehicle control 96 h post printing for 72 h. In situ fluorescent staining was conducted using the LIVE/DEAD Viability/Cytotoxicity Kit (cat. #L3224, ThermoFisher Scientific) following the manufacturer's instructions. Counterstaining for cell nuclei was performed using Hoechst 33342 (cat. #H1399, ThermoFisher Scientific). Briefly, cell media was removed, and cells were rinsed twice with 1× PBS followed by incubation with the staining cocktail for 30 min at 37°C and 5% CO_2_. Bioprinted cell models were imaged with a Stellaris SP8 Inverted Confocal System (Leica Biosystems, Nussloch, Germany) with sequential scanning using a 10× objective. Composite tile scans were generated utilising the ‘mosaic merge’ function in the Leica LAS X Navigator suite. Image resolution was fixed at 1024 × 1024 pixels and images were acquired at z‐steps of 5 μm at a total stack thickness of 150–200 μm. Resultant z‐stacks were merged into maximum‐intensity projections for analysis in open‐source FIJI (ImageJ) software [40]. To enhance contrast and reduce background noise, each fluorescent channel was uniformly processed with ‘Unsharp Mask’ (radius: 1 pixel, mask weight: 0.09) and ‘Median’ (radius: 2 pixels) filters, followed by conversion into binary masks via the ‘threshold’ function.

Live and dead cells were quantified based on Hoechst 33342‐positive nuclear counterstaining. To ensure accurate segmentation of densely‐packed nuclei, a distance transformation using the ‘Distance Map’ process was first applied to enhance the central region of each nucleus based on published methodology [41, 42]. The ‘Ultimate Points’ function was utilised to generate individual markers corresponding to this central region and the resultant image was used as ‘marker seeds’ for watershed segmentation using the ‘Marker‐Controlled Watershed’ function of the MorphoLibJ Plugin [43]. To identify live/dead nuclei, the ‘Image Calculator’ function using the logical ‘AND’ operation was applied to the binary masks of the nuclei and the live/dead channels, respectively, yielding binary masks containing live or dead nuclei only. Live or dead nuclei were subsequently counted using the ‘Analyse Particles’ function with a size threshold of 10μm^2^—*infinity* to exclude debris.

### 2.6 Clonogenic Cell Survival Assays

Clonogenic cell survival assays were conducted to determine longer‐term proliferative capacity of cells post treatment with ibrutinib, complementing MTS assays that determined short‐term cell viability post treatment with this drug. Cells were seeded into 6‐well plates at a density of 750 cells/well for OV207, 1000 cells/well for OVTOKO, and 2000 cells/well for OVISE, allowed to attach for 3–4 h and then treated with a final concentration range of 0.05–25 μM ibrutinib with 2‐fold dilutions for 72 h. After 7 days (OVTOKO and OV207) or 10 days (OVISE) post seeding, colony formation was assessed by fixation in 100% methanol for 20 min and staining with 0.5% *w/v* crystal violet in 25% *v/v* methanol for 5 min. Colonies of > 50 cells were counted using the GelCount imager (Oxford Optronix, Abingdon, England) and plating efficiency (PE) and survival fraction (SF) determined as previously described [44]. Cell survival was expressed relative to the DMSO vehicle control and dose response curves and AUC analyses were computed in GraphPad Prism v10.4.1 (GraphPad Software, MA, USA).

### 2.7 Statistical Analyses

Two‐sample *t*‐tests and one‐way ANOVA with Tukey's *post hoc* test for multiple comparisons were performed using GraphPad Prism v10.4.1 (GraphPad Software, MA, USA) unless otherwise stated. *p* < 0.05 were considered significant.

## 3 Results

### 3.1 Ibrutinib Inhibits Viability of OCCC Cells to a Greater Extent Than Non‐OCCC

**FIGURE 1:**
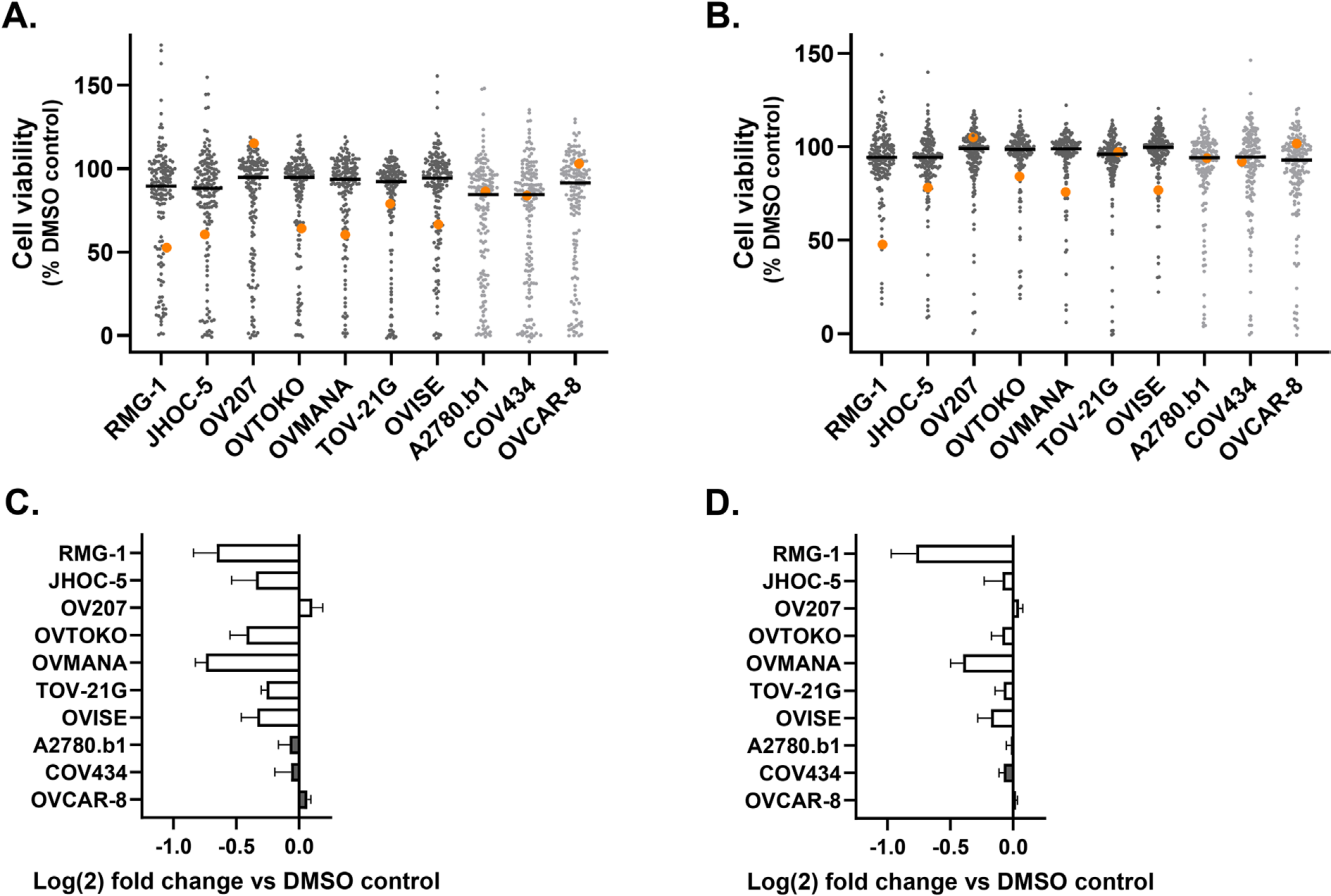
Screening OCCC and non‐OCCC cell lines with an epigenetic compound library. Cell viability as a % of DMSO control at (A) 5 μM and, (B) 0.5 μM of each of 160 compounds tested in triplicate replicates against 7 OCCC cell lines (RMG‐1, JHOC‐5, OV207, OVTOKO, OVMANA, TOV‐21G and OVISE) and 3 non‐OCCC cell lines (A2780.b1, COV434 and OVCAR‐8); median values indicated by a black bar. Drug 152 (ibrutinib) is indicated by an orange dot (*n* = 1) (C) 5 μM and, (D) 0.5 μM of ibrutinib treated OCCC and non‐OCCC cell lines shown as the mean ± S.E.M. (*n* = 3). OCCC cells are indicated by an open bar and non‐OCCC by a shaded bar.

The results of hypothesis generating primary screening for cell viability of all 7 of the OCCC cell lines and 3 non‐OCCC cell lines (A2780.b1, COV434 and OVCAR‐8) in 2D cultures against 160 drug compounds of the Tocris Epigenetics Library tested in triplicate at 0.5 and 5 μM is presented (Figure 1A,B). Heatmaps for all drugs tested in all cell lines at both concentrations are shown Figure S1. Drugs were triaged for further analyses based on a number of factors considered sequentially. Firstly, the preferential ability of the drug to inhibit viability in OCCC versus non‐OCCC cell lines by ≥ 10% was assessed, comparing the average viability across both groups. Secondly, the compounds needed to show the same trend across cell lines at both drug concentrations tested. The only drug that emerged following these criteria was the Bruton's Tyrosine Kinase (BTK) inhibitor ibrutinib, which showed greater efficacy in most OCCC compared with non‐OCCC cell lines at both drug concentrations tested. As this screen was conducted in biological singleton with three technical replicates, ibrutinib treatment of these cell lines was repeated at 5 μM and 0.5 μM in biological triplicate followed by cell viability analyses. With the exception of OV207, greater inhibition of cell viability was seen in all OCCC cell lines at both concentrations of ibrutinib relative to the non‐OCCC cell lines tested. Inhibition of cell viability was seen to a relatively smaller extent in the non‐OCCC cell lines A2780.b1 and COV434 compared with the majority of OCCC cell lines at both doses of ibrutinib (Figure 1C,D).

**FIGURE 2:**
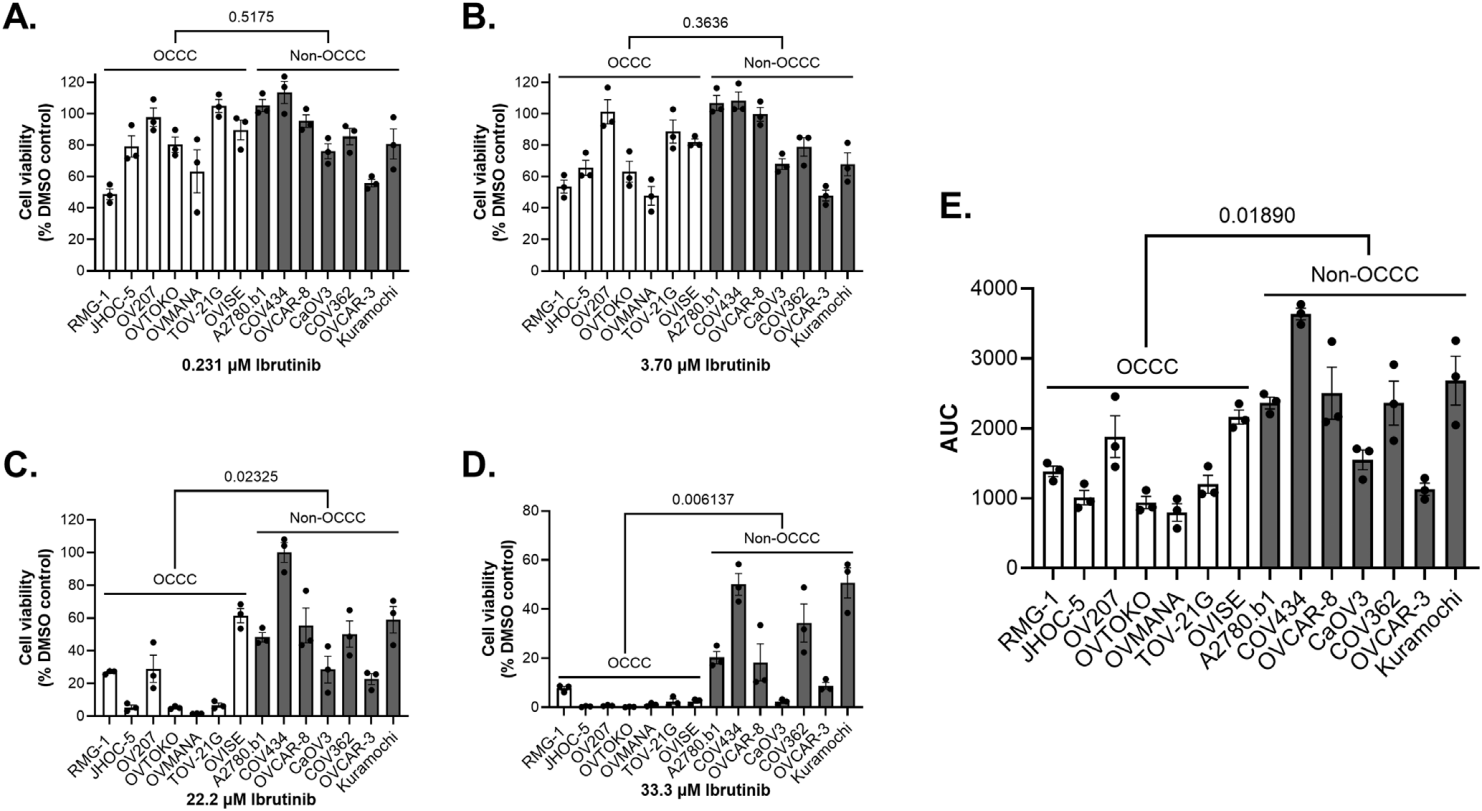
Treatment with ibrutinib decreases cell viability to a greater extent in OCCC cells compared to non‐OCCC. (A‐D) Bar graphs comparing cell viability of OCCC (RMG‐1, JHOC‐5, OV207, OVTOKO, OVMANA, TOV‐21G, and OVISE) and non‐OCCC (A2780.b1, COV434, OVCAR‐8, CaOV‐3, COV362, OVCAR3, and Kuramochi) cells following ibrutinib treatment relative to a DMSO control. Bars represent mean ± SEM (*n* = 3). Individual replicates are shown. OCCC are represented by an open bar, and non‐OCCC by a shaded bar. *p*‐values were calculated for the mean of OCCC versus non‐OCCC cell lines using an unpaired parametric *t*‐test, with *p*‐values recorded on the graphs. (E) Area‐under‐the‐curve (AUC) measurements show that ibrutinib significantly inhibits the viability of OCCC cells relative to non‐OCCC.

Next, we expanded the non‐OCCC cell line group to include additional HGSOC cell lines CaOV‐3, COV362, Kuramochi, OVCAR‐3, and OVCAR‐8. We then treated all cell lines with a serial dilution of ibrutinib (0.004–50 μM) and present data at selected doses (Figures 2A–D and S2) and as area‐under‐the‐curve (AUC) (Figure 2E). Statistically significant differences were observed in cell viability between OCCC and non‐OCCC cell lines treated with ≥ 22 μM of ibrutinib (Figure 2C,D). Grouping together measurements of cell viability presented as AUC, OCCC cell lines demonstrated greater sensitivity to ibrutinib compared to non‐OCCC cell lines (*p* = 0.0189, two‐sample *t*‐test, Figure 2E). There were some individual exceptions to this, including A2780.b1, which is known to carry both *ARID1A* and *ARID1B* mutations, that displayed similar sensitivity to ibrutinib as OCCC cell lines. The HGSOC cell lines CaOV‐3 and OVCAR‐3 also showed sensitivity to ibrutinib comparable to the OCCC cell line group. COV434, OVCAR‐8, and Kuramochi displayed lower sensitivities to ibrutinib (Figure 2E). RMG‐1 and other cell lines including OVISE displayed a small but reproducible biphasic response to ibrutinib Figure S2.

### 3.2 Ibrutinib Decreases Cell Viability of 3D Bioprinted OCCC Cells

**FIGURE 3:**
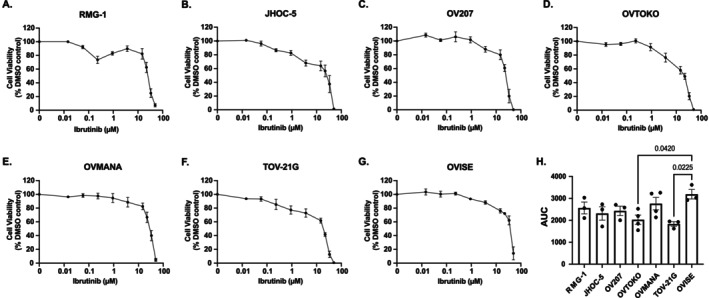
3D bioprinted OCCC cell models treated with ibrutinib. (A‐G) Dose response curves for ibrutinib‐treated 3D models of OCCC cell lines (A) RMG‐1, (B) JHOC‐5, (C) OV207, (D) OVTOKO, (E) OVMANA, (F) TOV‐21G, and (G) OVISE. Data represent mean cell viability ± SEM of *n* = 3–4 independent experiments, as a percentage of DMSO control versus the concentration of ibrutinib (μM) plotted on a log_10_ scale. (H) Column graph compares area‐under‐the‐curve (AUC; mean ± SEM) of 3D dose response curves. Points represent replicate experiments (*n* = 3–4). Analyses were undertaken using a one‐way ANOVA with Tukey's *post hoc* test for multiple comparisons. Significant *p*‐values are recorded on the graphs.

All OCCC cell lines underwent 3D bioprinting and treatment with ibrutinib as described, with data presented as dose response curves (Figure 3A–G and AUC Figure 3H). Statistically significant differences in sensitivity to ibrutinib between some OCCC cell lines were observed (Figure S3 and Figure 3H). The same reproducible biphasic response to ibrutinib observed in 2D cultures of RMG‐1 (Figure S2A) was also observed in 3D bioprinted cells (Figure 3A).

**FIGURE 4:**
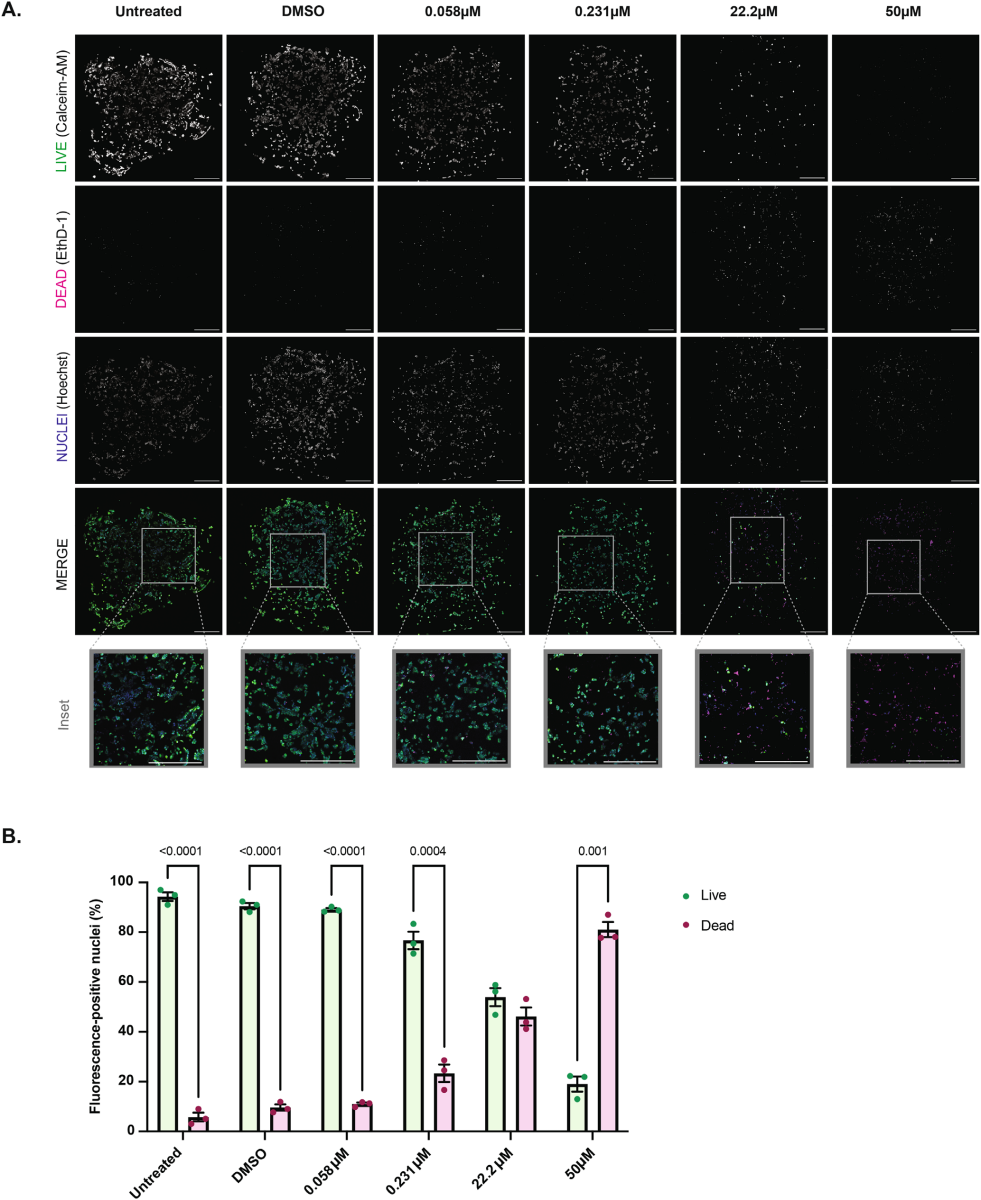
Ibrutinib exhibits cytotoxic effects on the 3D bioprinted OCCC cell line OVMANA. (A) Representative maximum z‐projection confocal micrographs with LIVE/DEAD Viability/Cytotoxicity (ThermoFisher Scientific, Australia) staining after 72 h incubation with ibrutinib, DMSO control or untreated media from *n* = 3 independent replicates of OVMANA 3D culture models. Live cells are indicated by positive Calcein‐AM staining (green), dead cells with ethidium homodimer‐1 (EthD‐1; magenta) and cell nuclei with Hoechst 33342 (blue). Regions indicated within grey boundaries are enlarged in corresponding inset images. Scale bar = 500 μm. (B) Column graphs show live (green) and dead (magenta) cells as a percentage (± SEM) of total bioprinted cells (via Hoechst 33342‐positive nuclear counterstaining) following treatment with select doses of ibrutinib. Binary masks of individual fluorescent channels were generated in open‐source FIJI (Image J) software. Live and dead nuclei were subsequently quantified with the ‘Analyse Particles’ function and computed in Microsoft Excel Software (Microsoft, USA). Statistical analysis was performed using GraphPad Prism v10.4.1 software (GraphPad Software, USA). Data represents 3 independent experimental replicates as indicated by data points within each column. Adjusted *p*‐values were calculated using a one‐way ANOVA with Dunnett's *post hoc* test comparing ibrutinib treatment groups to the DMSO vehicle control, with statistically significant adjusted *p*‐values recorded on the graphs.

Next, the ARID1A WT cell line RMG‐1 and mutant cell line OVMANA underwent fluorescent imaging to detect live and dead cells in response to a dose curve of ibrutinib (Figures 4A and S4A). Interestingly, these cell lines displayed similar responses to ibrutinib when cultured under these conditions, with around 20% of cells non‐viable in each culture at 0.231 μM ibrutinib and over 80% of cells non‐viable in both cell lines at 50 μM (Figures 4B and S4B).

### 3.3 Ibrutinib Inhibits Clonogenic Cell Survival of OCCC Cells

**FIGURE 5:**
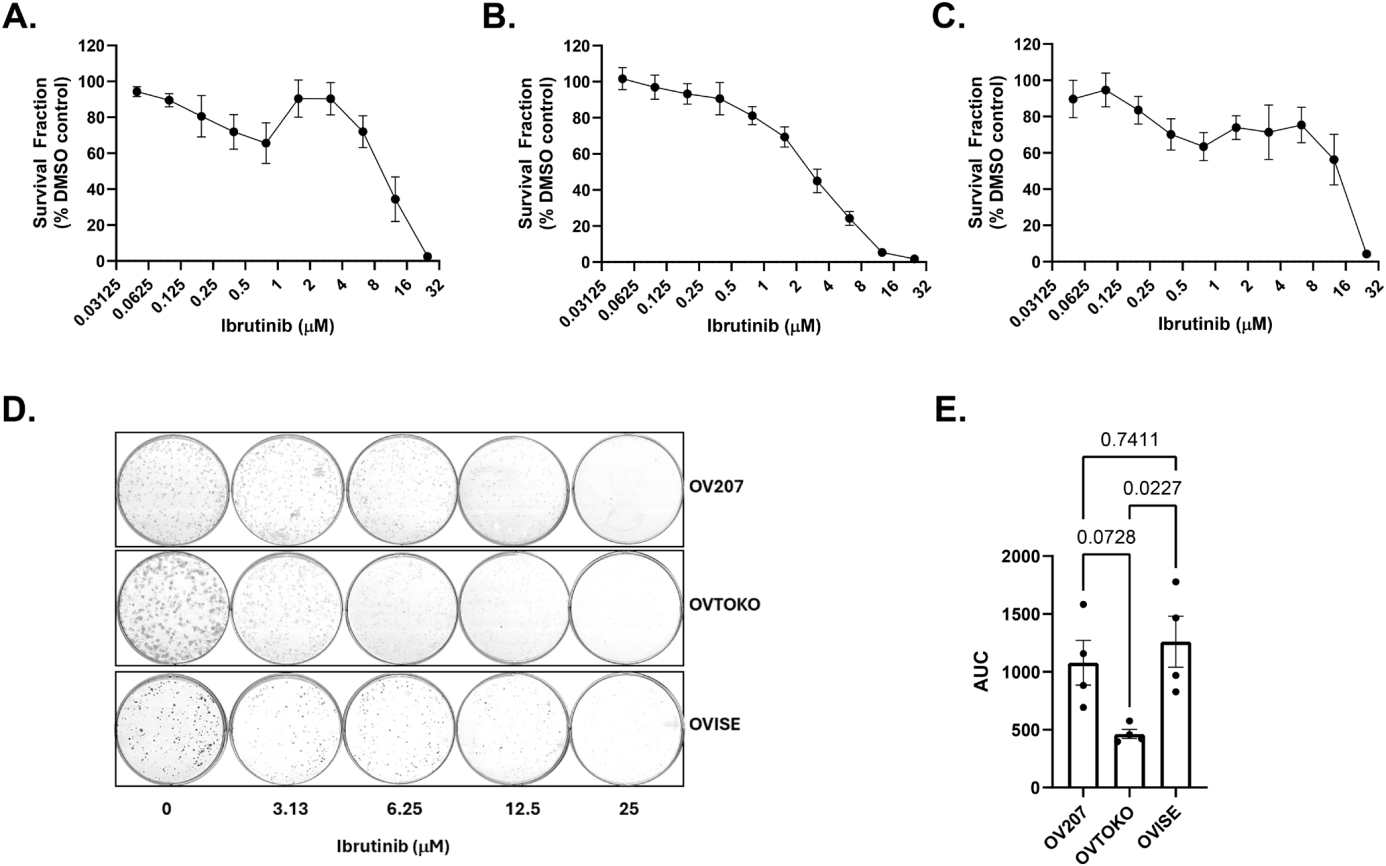
Ibrutinib inhibits clonogenic cell survival in OCCC cell line models. Dose ranges of ibrutinib between 0.05–25 μM (two‐fold dilutions) were tested on (A) OV207, (B) OVTOKO and (C) OVISE, with images (D) shown from selected concentrations and data presented as AUC (E). Bars represent mean ± SEM (*n* = 4), with individual replicates shown. Statistical significance was determined using a one‐way ANOVA with Tukey's *post hoc* test for multiple comparisons, and *p*‐values are recorded on the graphs.

In order to complement short‐term cell viability assays, we undertook clonogenic cell survival assays to investigate longer‐term recovery and proliferative capacity of OCCC cells post‐treatment with ibrutinib. Not all OCCC cell lines proved suitable for this assay, leading to a focus on OVTOKO, OV207 and OVISE. Ibrutinib inhibited clonogenic cell survival in all cell lines analysed (Figure 5).

### 3.4 Ibrutinib Decreases Levels of Phosphorylated AKT in a Dose Dependent Manner

**FIGURE 6:**
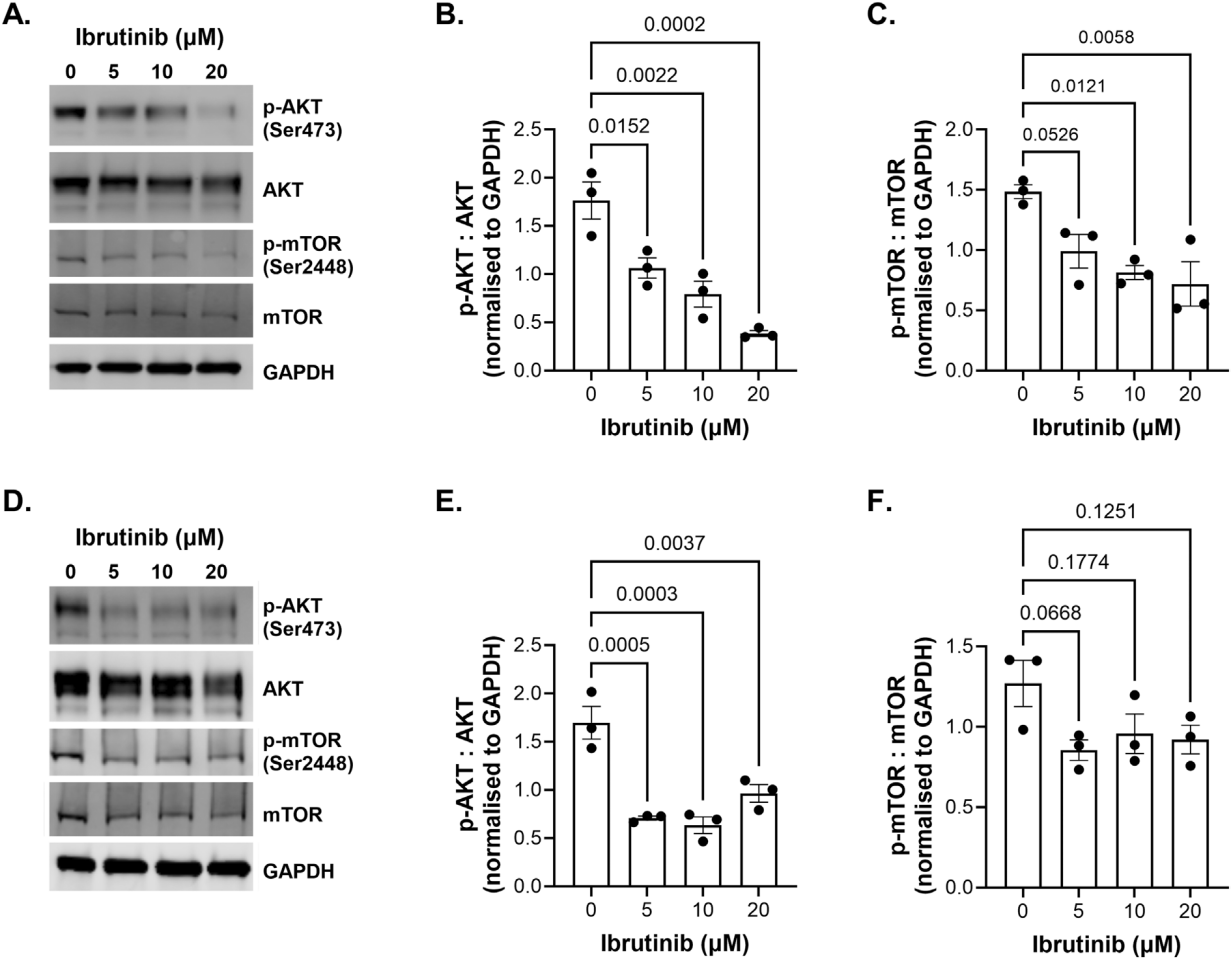
Ibrutinib inhibits PI3K/AKT/mTOR signalling in OVTOKO and OVMANA cell lines. Analyses of OVTOKO (A) Western blot showing p‐AKT (Ser473)/total AKT and p‐mTOR(Ser2448)/total mTOR after 48 h treatment with ibrutinib (0, 5, 10 and 20 μM). (B) Quantification of p‐AKT to total AKT normalised to GAPDH and (C) Quantification of p‐mTOR to total mTOR normalised to GAPDH; Analyses of OVMANA showing (D) Western blot showing p‐AKT (Ser473)/total AKT and p‐mTOR(Ser2448)/total mTOR after 48 h treatment with ibrutinib (0, 5, 10 and 20 μM). (E) Quantification of p‐AKT to total AKT normalised to GAPDH and (F) Quantification of p‐mTOR to total mTOR normalised to GAPDH. Data represents 3 independent experimental replicates shown as the mean ± SEM. *p*‐values were calculated using a one‐way ANOVA with Tukey's *post hoc* test for multiple comparisons and indicated on the graphs.

Lastly, we sought to determine whether ibrutinib functioned to inhibit the PI3K/AKT/mTOR cell survival pathway in OCCC cells. Treatment of these cell lines over 48 h with 5, 10 and 20 μM of ibrutinib showed significant decreases of phosphorylated AKT in OVTOKO and OVMANA cell lines; however, significant decreases in phosphorylated mTOR were only observed for OVTOKO cells (Figure 6 and Figure S6). This was not; however, the case for all cell lines tested. While TOV‐21G cells showed decreased levels of phosphorylated AKT at the higher ibrutinib doses (10 and 20 μM), phosphorylated AKT and phosphorylated mTOR remained largely unchanged in response to different doses of ibrutinib in RMG‐1 and TOV‐21G cell lines (Figures S5 and S6). This suggests that ibrutinib may not inhibit PI3K/AKT/mTOR signalling in all cases of OCCC.

## 4 Discussion

OCCC is characterised by chemoresistance, particularly upon relapse where the vast majority of patients do not respond to standard‐of‐care platinum‐taxol‐based chemotherapy. While clinical trials for immunotherapy drugs show some promise [20, 21, 22, 23, 24], OCCC remains a difficult‐to‐treat malignancy. We screened an epigenetic compound library against both OCCC and non‐OCCC cell line models with the goal of generating hypotheses regarding a drug, or multiple drugs, to specifically target OCCC. This screen identified only one compound, the BTK inhibitor ibrutinib, as effective in specifically decreasing the viability of OCCC cells at both concentrations tested and relative to non‐OCCC cell lines. The majority of OCCC cells showed a greater sensitivity to ibrutinib than non‐OCCC cells. Some OCCC cell lines, including RMG‐1 and OVISE, displayed a mild but reproducible biphasic response to ibrutinib, possibly indicating off‐target effects at higher concentrations that promote cell survival, and/or reflecting the co‐existence of different subpopulations with different drug sensitivities within the same cell line. Responses to ibrutinib were observed in both short‐term viability assays and longer‐term cell survival assays in OCCC cell line models.

A study of a cohort of 50 primary ovarian tumours of mixed pathologies (including three OCCCs) reported that BTK was present in most cases, and that higher levels were associated with lower survival rates [45]. BTK and its isoforms are also seen in other solid tumours including colorectal, breast, and prostate cancers, as well as glioblastoma, non‐small cell lung cancers, and others [46, 47, 48, 49]. These and additional studies highlight BTK, not only as a potential prognostic marker, but as a possible therapeutic target in solid malignancies including OCCC. Of interest, ibrutinib was tested in a murine model of syngeneic endometrial transplantation and shown to inhibit the development of endometriosis‐like lesions [50].

Ibrutinib is a first generation, irreversible small molecule inhibitor of BTK sold under the tradename Imbruvica. This drug is FDA approved for the treatment of B cell‐related disorders including chronic lymphocytic leukaemia (CLL), small lymphocytic lymphoma (SLL), graft‐versus‐host disease (GVHD), mantle cell lymphoma (MCL), marginal zone lymphoma (MZL), and Waldenstrom macroglobulinaemia (WM) (reviewed in [51]). Of significant interest, ibrutinib was used to treat CLL in a patient who had the incidental finding of a low‐grade serous ovarian cancer (LGSOC). This patient received standard adjuvant platinum‐based chemotherapy to treat their ovarian cancer; however, the ovarian cancer recurred and levels of the ovarian cancer biomarker CA‐125 increased. In response to recurrence of their CLL, oral ibrutinib was administered (140 mg, alternating 2 times, then 3 times daily), resulting in decreased CA‐125 that normalised after 12 months of therapy with no clinical signs of ovarian cancer. Cessation of ibrutinib saw increasing CA‐125 after a six‐week period. Subsequent analyses of their ovarian tumour showed variants in *KRAS* (typically associated with low‐grade ovarian tumours), *MUTYH* and *ARID1A* [52].

Ibrutinib has also been administered off‐label as an oral monotherapy (420 mg daily) to a patient with platinum‐resistant advanced LGSOC. It was identified as the preferred candidate from a drug‐sensitivity panel implemented on a primary organoid culture of the LGSOC. Whole exome sequencing revealed a pathogenic mutation in *CHEK2* as well as variants of unknown significance in *AKT1*, *IRS2*, *MLL2*, *MSH3*, *MUTYH*, and *NOTCH3*. Normalisation of this patient's CA‐125 levels and dramatic clinical improvement were seen over a 65‐week period taking this drug before CA‐125 levels again began to rise and an alternative drug was administered [53]. It will be interesting to determine the signalling pathways affected in LGSOC in response to ibrutinib.

BTK is a non‐receptor tyrosine kinase, and upon its phosphorylation activates key cancer survival pathways including PI3K/AKT/mTOR, RAS‐MAPK, and NF‐κB [54]. Of note, activating mutations of *PIK3CA* that encodes one of the catalytic subunits of PI3K frequently occur alongside mutation of *ARID1A* as an early event in OCCC [31, 32, 55], together driving an inflammatory environment and upregulating the PI3K/AKT/mTOR pathway, making this pathway of particular interest in these tumours [56]. We have shown that ibrutinib suppresses phosphorylated AKT and mTOR in some, but not all, OCCC cell lines. It is possible that in RMG‐1 and TOV‐21G where ibrutinib does not appear to act on AKT/mTOR signalling, it may act via alternative pathways including RAS‐MAPK or NF‐κB. Taken together, our data highlights the potential of ibrutinib for the treatment of OCCC.

As a first‐generation BTK inhibitor, ibrutinib selectively and covalently binds to cysteine 481 in the ATP‐binding site of BTK, thereby inhibiting subsequent phosphorylation of BTK; however, it can also bind with different affinities to other kinases that have a similar cysteine residue in their ATP‐binding sites with varying affinities, including members of the EGFR family (EGFR, ErbB2/HER2 and ErbB4/HER4), BLK and JAK3, as well as TFK family members (ITK, TEC, BMX and RLK/TXK) [57]. Off‐target kinase, or other, binding of ibrutinib may contribute to toxicities [57]. Second and third generation BTK inhibitors have reduced off‐target affinities to other kinases, with the third generation BTK inhibitor pirtobrutinib being the first reversible BTK inhibitor approved by the FDA to treat CLL/SLL and MCL [47, 51, 58, 59, 60, 61]. Additionally, PROTAC (proteolysis‐targeting chimera) BTK degraders are currently in clinical trials for B cell malignancies and may be considered in the future to treat solid tumours [59].

This study does have some limitations. The primary drug screen was performed with hypothesis generating goals, relying heavily on downstream validation and additional experiments as described. This may have limited discoveries other than ibrutinib being revealed from the initial screen. Furthermore, we have not determined whether the primary effect of ibrutinib in OCCC models is on BTK or other known targets as mentioned above. Lastly, while this study incorporates a spectrum of ovarian cancer subtypes including HGSOC, endometrioid carcinoma, and the exceedingly rare SCCOHT, future investigations using primary patient‐derived models (subject to tissue availability) may further strengthen the translational potential of ibrutinib. In summary, we have shown that ibrutinib displays greater efficacy against OCCC compared to non‐OCCC cell lines. As future generations of reversible and irreversible BTK inhibitors are developed with greater specificity, as well as BTK degraders, opportunities will arise to bring the success of these drugs in treating B cell disorders into the field of solid tumours, including for chemoresistant malignancies such as OCCC.

## Author Contributions

**Yue Ma:** investigation, methodology, data curation, visualisation, writing – original draft preparation, writing – reviewing and editing. **Kristie‐Ann Dickson:** investigation, methodology, data curation, visualisation, supervision, writing – original draft preparation, writing – reviewing and editing. **Farhana A. Sarker:** investigation, methodology, data curation, visualisation, writing – original draft preparation, writing – reviewing and editing. **Amani Alghalayini:** investigation, data curation, writing – reviewing and editing. **Natisha R. Field:** investigation, writing – reviewing and editing. **Tao Xie:** investigation, writing – reviewing and editing. **Tali S. Skipper:** visualisation, writing – reviewing and editing. **Anastasia Karafotias:** methodology, data curation, visualisation, writing – reviewing and editing. **Sarina Briscas:** investigation, writing – reviewing and editing. **Christine Yee:** methodology, writing – reviewing and editing. **Caroline E. Ford:** conceptualisation, writing – reviewing and editing. **Nikola A. Bowden:** funding acquisition, conceptualisation, writing – reviewing and editing. **Nham Tran:** supervision, writing – reviewing and editing. **Deborah J. Marsh:** conceptualisation, funding acquisition, project administration, supervision, visualisation, writing – original draft preparation, writing – reviewing and editing.

## Funding

This work was supported by the National Health and Medical Research Council, 2019296. Chinese Scholarship Council, Australian Commonwealth Government through the Department of Education and Training. Vanessa McGuigan Memorial Hunter Medical Research Institute Fellowship.

## Conflicts of Interest

The authors declare no conflicts of interest.

## Supporting information


**Figure S1:** Heatmaps showing cell viability of 7 OCCC (RMG‐1, JHOC‐5, OV207, OVTOKO, OVMANA, TOV‐21G, OVISE) and 3 non‐OCCC (A2780.b1, COV434, OVCAR‐8) cell lines treated in triplicate replicates with (A) 5 and (B) 0.5 μM of drugs in the Tocriscreen Epigenetics Library. Drug 152 is ibrutinib.

**Figure S2:** Dose response curves of 7 OCCC (A‐G; RMG‐1, JHOC‐5, OV207, OVTOKO, OVMANA, TOV‐21G, OVISE) and 7 non‐OCCC (H‐N; A2780.b1, COV434, OVCAR‐8, CaOV‐3, COV362, Kuramochi, OVCAR‐3) cell lines cultured in 2D and treated with ibrutinib. Curves are plotted as a concentration of ibrutinib (μM) on a log_10_ scale against cell viability as a percentage of DMSO control represented as the mean ± SEM (*n* = 3).

**Figure S3:** 3D bioprinted OCCC cell models treated with ibrutinib. Column graphs show mean ± SEM for cell viability of 3D bioprinted OCCC cell lines as a percentage of DMSO vehicle control following treatment with ibrutinib at concentrations over a dose curve (A) 0.015 μM, (B) 0.058 μM, (C) 0.231 μM, (D) 0.926 μM, (E) 3.7 μM, (F) 14.8 μM, (G) 22.2 μM and (H) 33.3 μM. Data reflects *n* = 3–4 independent experiments per OCCC cell line indicated by individual data points within each column. Data was analysed using a one‐way ANOVA with Tukey's *post hoc* test for multiple comparisons.

**Figure S4:** Ibrutinib exhibits cytotoxic effects on 3D bioprinted OCCC cell line RMG‐1. (A) Representative maximum z‐projection confocal micrographs with LIVE/DEAD Viability/Cytotoxicity (ThermoFisher Scientific, Australia) staining after 72 h incubation with ibrutinib, DMSO control or untreated media from *n* = 3 independent replicates of RMG‐1 3D culture models. Live cells are indicated by positive Calcein‐AM staining (green), dead cells with ethidium homodimer‐1 (EthD‐1; magenta) and cell nuclei with Hoechst 33342 (blue). Regions indicated within grey boundaries are enlarged in corresponding inset images. Scale bar = 500 μm. (B) Column graphs show live (green) and dead (magenta) cells as a percentage (± SEM) of total bioprinted cells (via Hoechst 33342‐positive nuclear counterstaining) following treatment with select doses of ibrutinib. Binary masks of individual fluorescent channels were generated in open‐source FIJI (Image J) software. Live and dead nuclei were subsequently quantified with the ‘Analyse Particles’ function and computed in Microsoft Excel Software (Microsoft, USA). Statistical analysis was performed using GraphPad Prism v10.4.1 software (GraphPad Software, USA). Data represents 3 independent experimental replicates as indicated by data points within each column. Adjusted *p*‐values were calculated using a one‐way ANOVA with Dunnett's *post hoc* test comparing ibrutinib treatment groups to the DMSO vehicle control, with statistically significant adjusted *p*‐values recorded on the graphs.

**Figure S5:** Ibrutinib does not inhibit PI3K/AKT/mTOR signalling in RMG‐1 and TOV‐21G cell lines. Analyses of RMG‐1 showing (A) Western blot showing p‐AKT (Ser473)/total AKT and p‐mTOR(Ser2448)/total mTOR after 48 h treatment with ibrutinib (0, 5, 10 and 20 μM). (B) Quantification of p‐AKT to total AKT normalised to GAPDH and, (C) Quantification of p‐mTOR to total mTOR normalised to GAPDH; Analyses of TOV‐21G showing (D) Western blot showing p‐AKT (Ser473)/total AKT and p‐mTOR (Ser2448)/total mTOR after 48 h treatment with ibrutinib (0, 5, 10 and 20 μM). (E) Quantification of p‐AKT to total AKT normalised to GAPDH and, (F) Quantification of p‐mTOR to total mTOR normalised to GAPDH. Data represents 3 independent experimental replicates shown as the mean ± SEM. *p*‐values were calculated using a one‐way ANOVA with Tukey's *post hoc* test for multiple comparisons.

**Figure S6:** Raw fluorescent images of western blots showing protein bands detected by NIR700 (near infrared 700 nm) signal (green) scanned on the Odyssey CLX instrument (LiCOR). (A) OVTOKO (lanes 1–4 replicate 1; lanes 5–8 replicate 2; lanes 9–12 replicate 3), (B) OVMANA (lanes 1–4 replicate 1; lanes 5–8 replicate 2), (C) TOV‐21G (lanes 1–4 replicate 1; lanes 5–8 replicate 2) and RMG‐1 (lanes 9–12 replicate 1), (D) RMG‐1 (lanes 1–4 replicate 2; lanes 5–8 replicate 3; lanes 9–12 replicate 4) and (E) OVMANA (lanes 1–4 replicate 3) and TOV‐21G (lanes 5–8 replicate 3). For all blots, vehicle control samples were loaded into lanes 1, 5 and 9; 5 μM ibrutinib treated samples into lanes 2, 6 and 10; 10 μM ibrutinib treated samples into lanes 3, 7 and 11; and 20 μM ibrutinib treated samples into lanes 4, 8 and 12. (F) Licor Chameleon NIR marker showing fluorescent proteins of indicated size (red, green or yellow) with a blue dotted line indicating where nitrocellulose membranes were cut in order to probe with specific antibodies. In panels (A‐E), the upper panels show 3 membrane pieces probed with p‐mTOR (~289 kDa), p‐AKT (~60 kDa) and GAPDH (~37 kDa), and the lower panels show 3 membrane pieces probed with mTOR (~289 kDa), AKT (~60 kDa) and GAPDH (~37 kDa). ^ Indicates a high brightness setting for low abundant proteins. ^^ Indicates a low brightness setting for high abundant proteins.

## Data Availability

The data that support the findings of this study are available from the corresponding author upon reasonable request.
