# Supplementary figures and images for: Epigenetic Compound Library Screen Identifies Ibrutinib as an Inhibitor of Ovarian Clear Cell Carcinoma Viability
Source: Cancer Med. 2026 Apr 8;15(4):e71795. doi: 10.1002/cam4.71795 (PMC13062270; doi:10.1002/cam4.71795)

A.

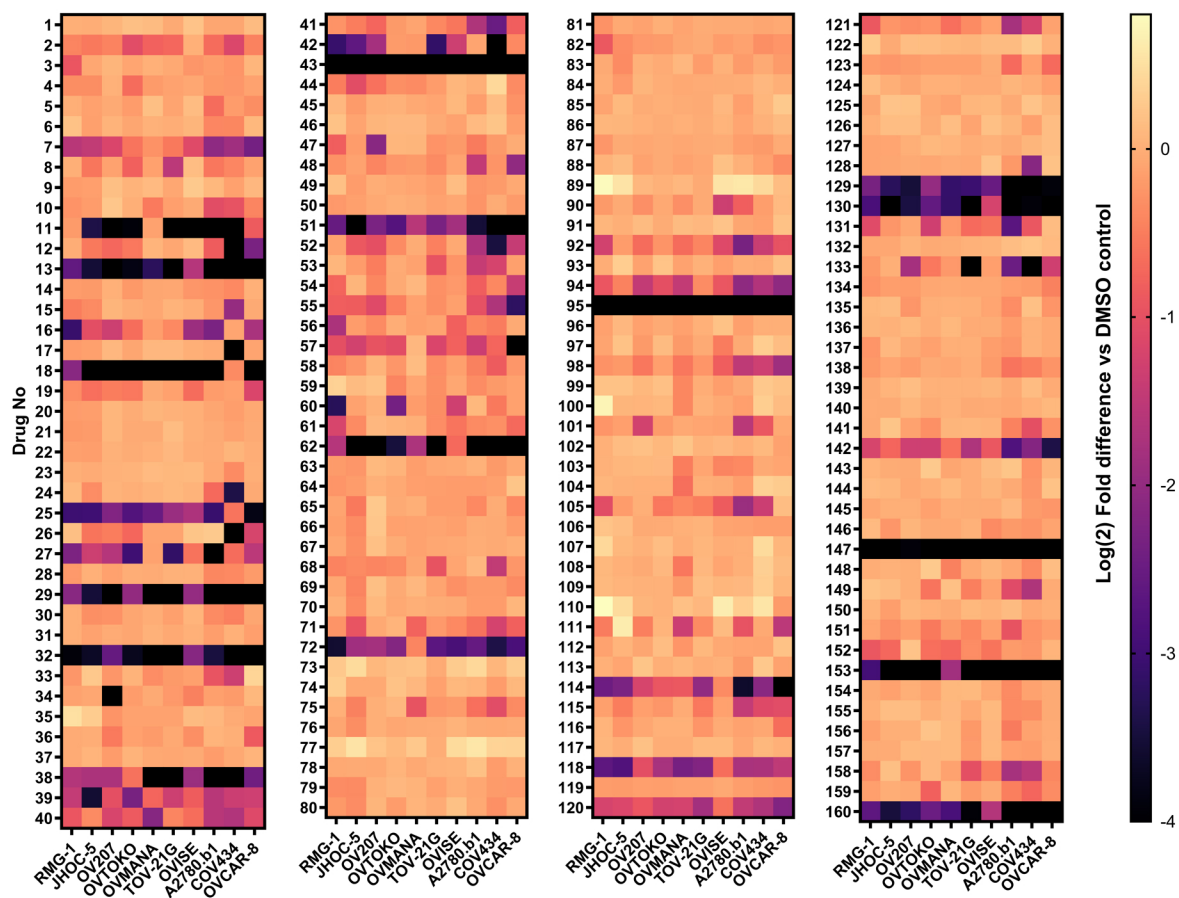

B.

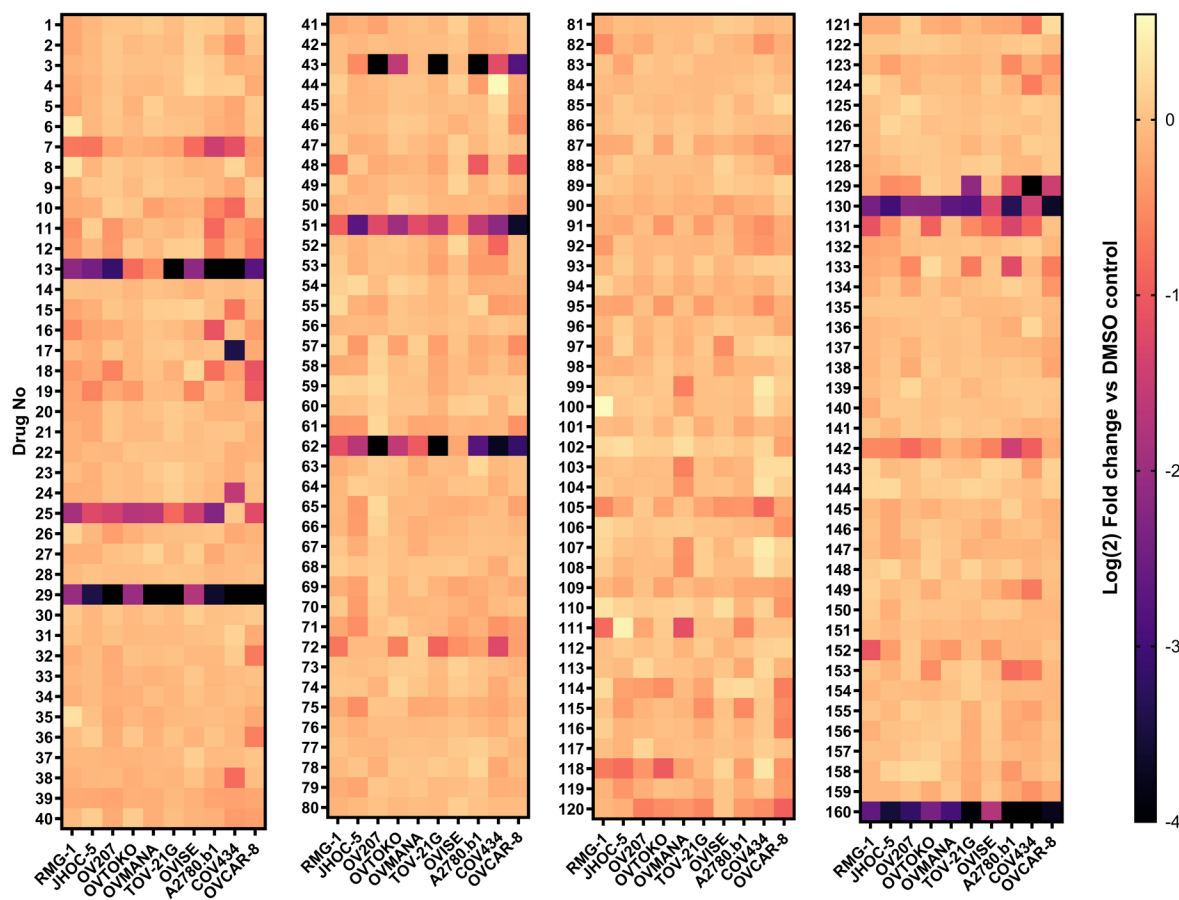

Supplement: Supplementary file 1 — Figure S1: Heatmaps showing cell viability of 7 OCCC (RMG‐1, JHOC‐5, OV207, OVTOKO, OVMANA, TOV‐21G, OVISE) and 3 non‐OCCC (A2780.b1, COV434, OVCAR‐8) cell lines treated in triplicate replicates with (A) 5 and (B) 0.5 μM of drugs in the Tocriscreen Epigenetics Library. Drug 152 is ibrutinib. [file CAM4-15-e71795-s005.pdf]

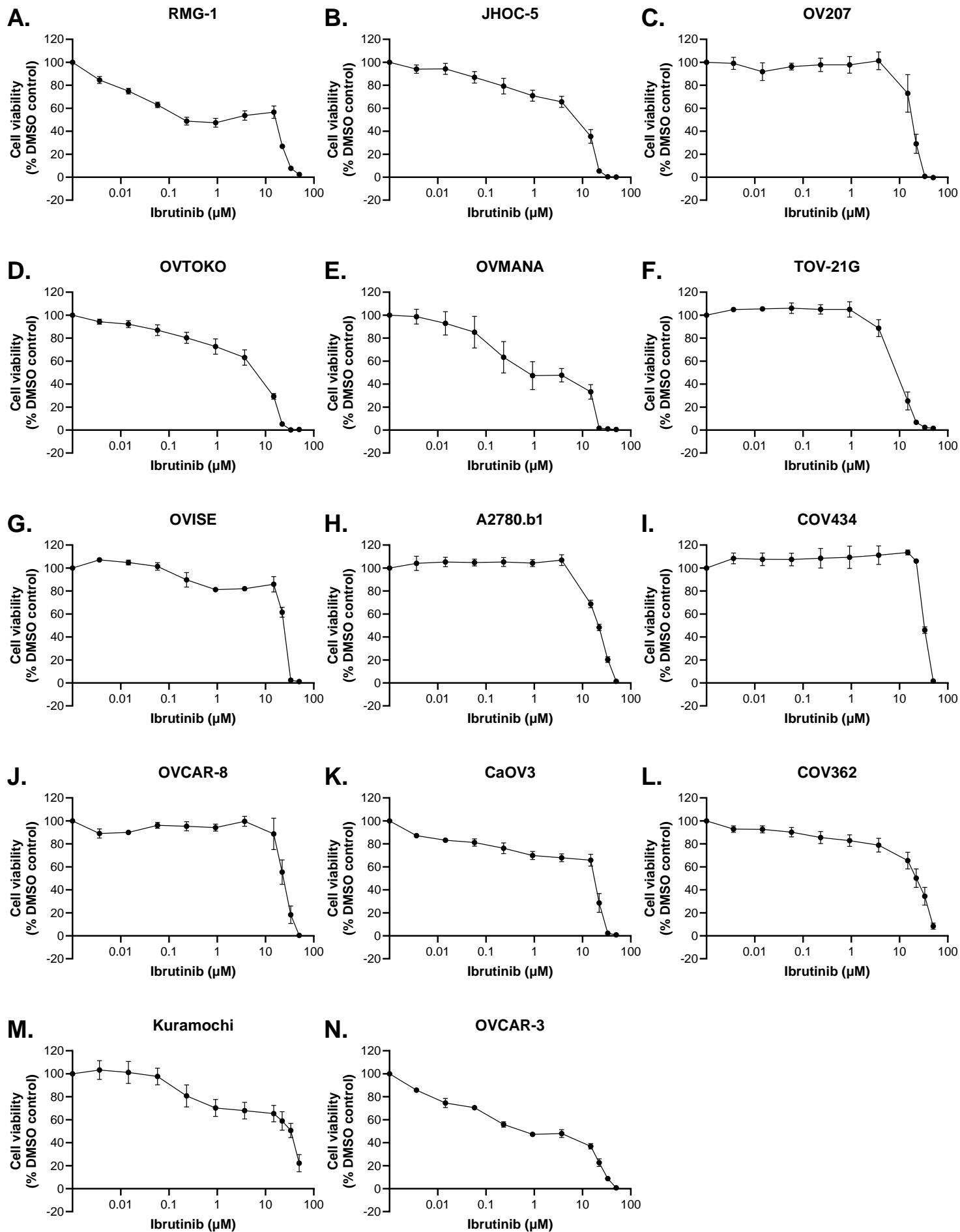

Supplement: Supplementary file 2 — Figure S2: Dose response curves of 7 OCCC (A‐G; RMG‐1, JHOC‐5, OV207, OVTOKO, OVMANA, TOV‐21G, OVISE) and 7 non‐OCCC (H‐N; A2780.b1, COV434, OVCAR‐8, CaOV‐3, COV362, Kuramochi, OVCAR‐3) cell lines cultured in 2D and treated with ibrutinib. Curves are plotted as a concentration of ibrutinib (μM) on a log10 scale against cell viability as a percentage of DMSO control represented as the mean ± SEM (n = 3). [file CAM4-15-e71795-s003.pdf]

A.

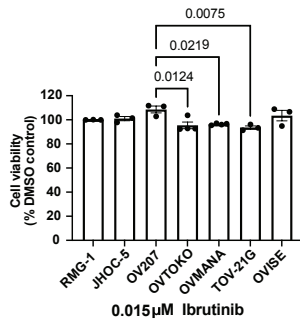

B.

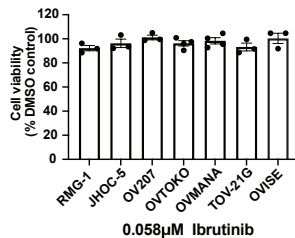

C.

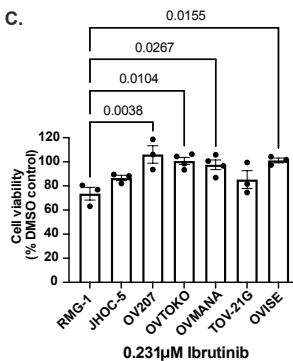

D.

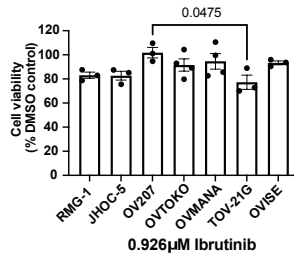

E.

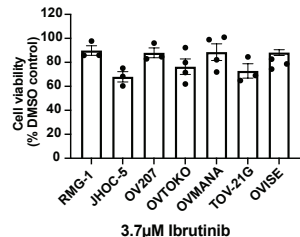

F.

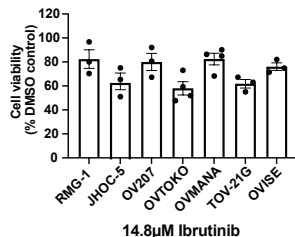

G.

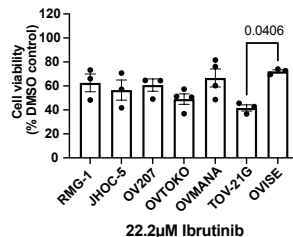

H.

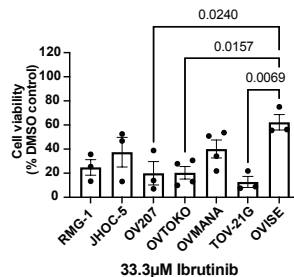

Supplement: Supplementary file 3 — Figure S3: 3D bioprinted OCCC cell models treated with ibrutinib. Column graphs show mean ± SEM for cell viability of 3D bioprinted OCCC cell lines as a percentage of DMSO vehicle control following treatment with ibrutinib at concentrations over a dose curve (A) 0.015 μM, (B) 0.058 μM, (C) 0.231 μM, (D) 0.926 μM, (E) 3.7 μM, (F) 14.8 μM, (G) 22.2 μM and (H) 33.3 μM. Data reflects n = 3–4 independent experiments per OCCC cell line indicated by individual data points within each column. Data was analysed using a one‐way ANOVA with Tukey's post hoc test for multiple comparisons. [file CAM4-15-e71795-s004.pdf]

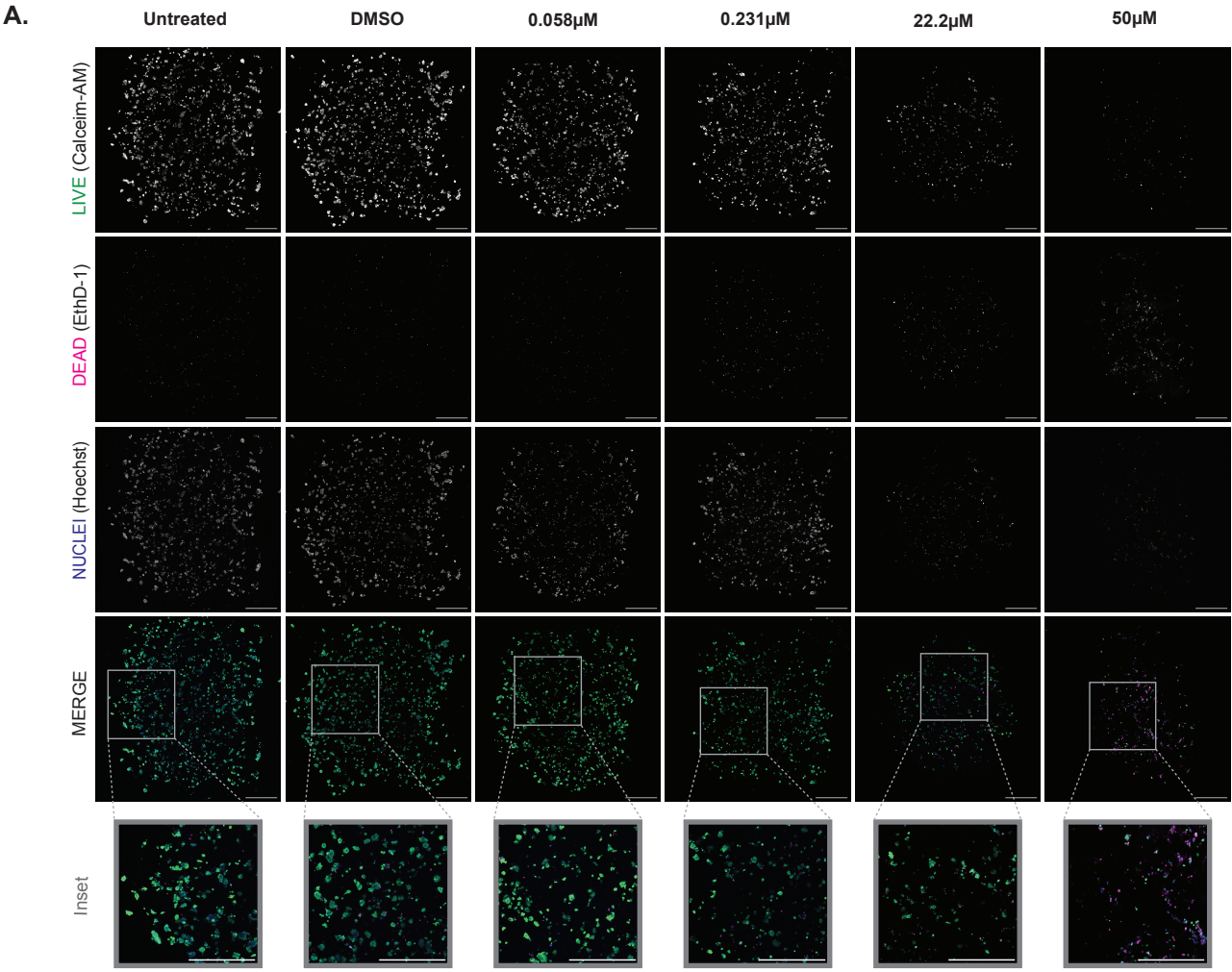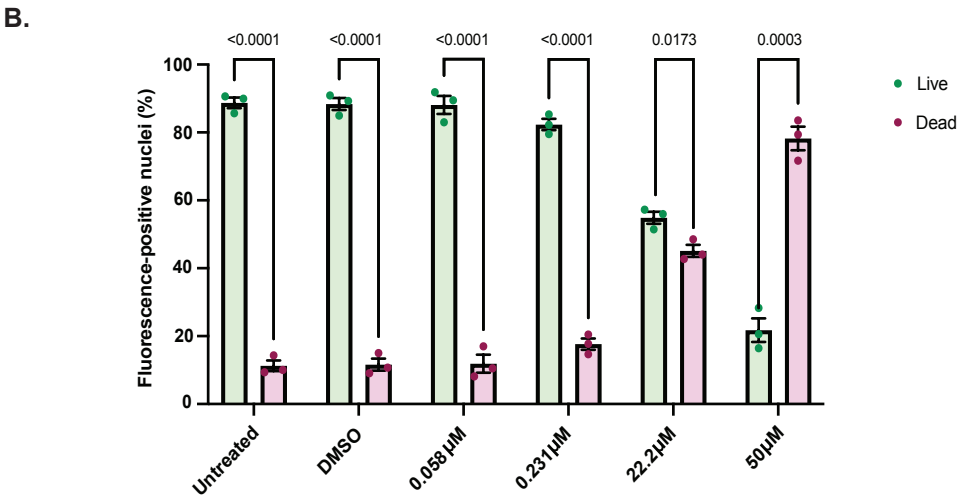

Supplement: Supplementary file 4 — Figure S4: Ibrutinib exhibits cytotoxic effects on 3D bioprinted OCCC cell line RMG‐1. (A) Representative maximum z‐projection confocal micrographs with LIVE/DEAD Viability/Cytotoxicity (ThermoFisher Scientific, Australia) staining after 72 h incubation with ibrutinib, DMSO control or untreated media from n = 3 independent replicates of RMG‐1 3D culture models. Live cells are indicated by positive Calcein‐AM staining (green), dead cells with ethidium homodimer‐1 (EthD‐1; magenta) and cell nuclei with Hoechst 33342 (blue). Regions indicated within grey boundaries are enlarged in corresponding inset images. Scale bar = 500 μm. (B) Column graphs show live (green) and dead (magenta) cells as a percentage (± SEM) of total bioprinted cells (via Hoechst 33342‐positive nuclear counterstaining) following treatment with select doses of ibrutinib. Binary masks of individual fluorescent channels were generated in open‐source FIJI (Image J) software. Live and dead nuclei were subsequently quantified with the ‘Analyse Particles’ function and computed in Microsoft Excel Software (Microsoft, USA). Statistical analysis was performed using GraphPad Prism v10.4.1 software (GraphPad Software, USA). Data represents 3 independent experimental replicates as indicated by data points within each column. Adjusted p‐values were calculated using a one‐way ANOVA with Dunnett's post hoc test comparing ibrutinib treatment groups to the DMSO vehicle control, with statistically significant adjusted p‐values recorded on the graphs. [file CAM4-15-e71795-s002.pdf]

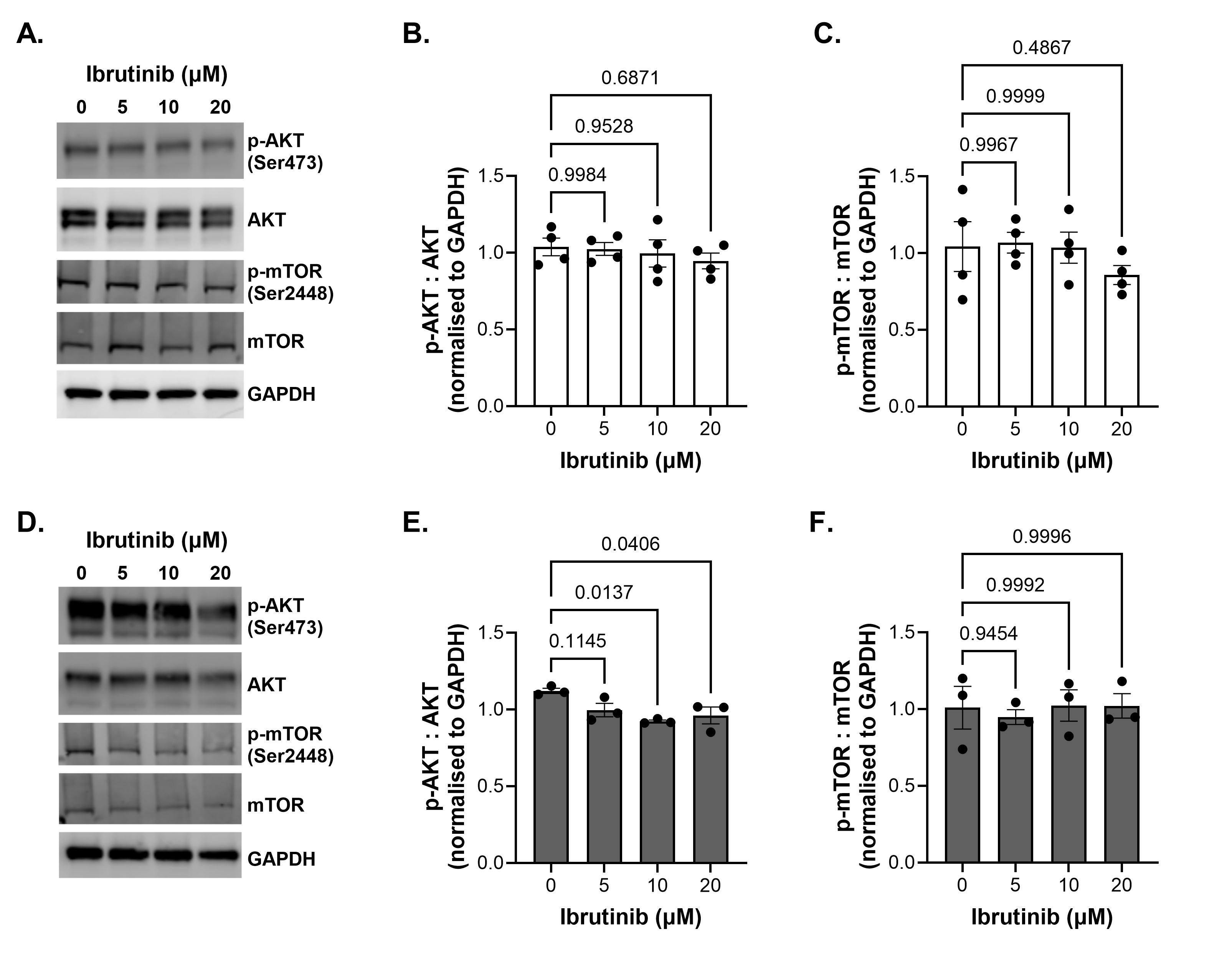

Supplement: Supplementary file 5 — Figure S5: Ibrutinib does not inhibit PI3K/AKT/mTOR signalling in RMG‐1 and TOV‐21G cell lines. Analyses of RMG‐1 showing (A) Western blot showing p‐AKT (Ser473)/total AKT and p‐mTOR(Ser2448)/total mTOR after 48 h treatment with ibrutinib (0, 5, 10 and 20 μM). (B) Quantification of p‐AKT to total AKT normalised to GAPDH and, (C) Quantification of p‐mTOR to total mTOR normalised to GAPDH; Analyses of TOV‐21G showing (D) Western blot showing p‐AKT (Ser473)/total AKT and p‐mTOR (Ser2448)/total mTOR after 48 h treatment with ibrutinib (0, 5, 10 and 20 μM). (E) Quantification of p‐AKT to total AKT normalised to GAPDH and, (F) Quantification of p‐mTOR to total mTOR normalised to GAPDH. Data represents 3 independent experimental replicates shown as the mean ± SEM. p‐values were calculated using a one‐way ANOVA with Tukey's post hoc test for multiple comparisons. [file CAM4-15-e71795-s001.tif]

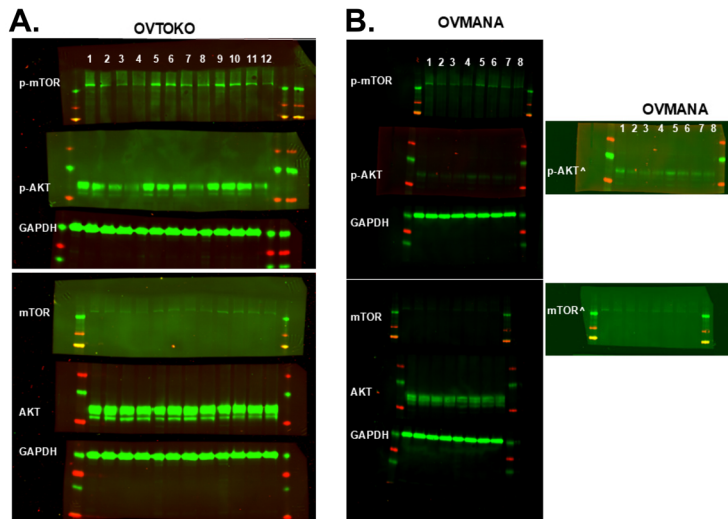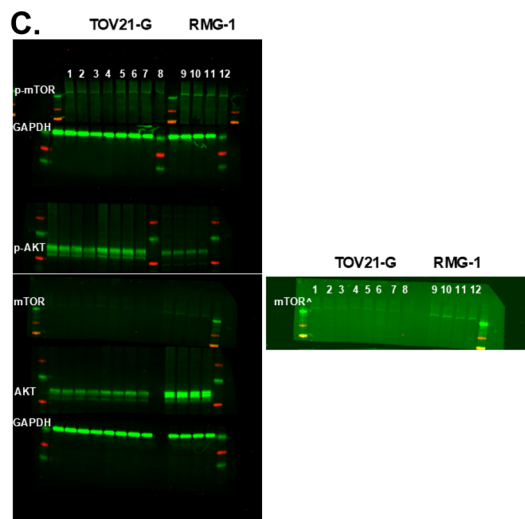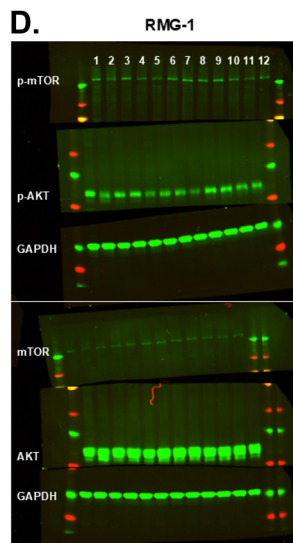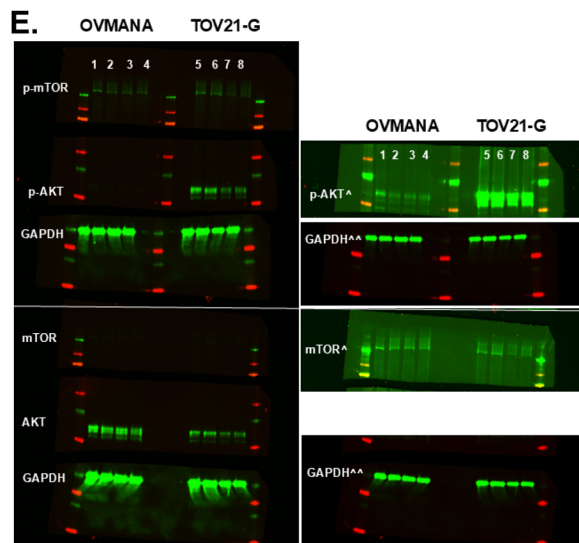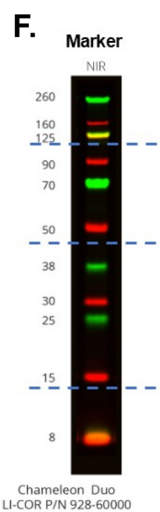

Supplement: Supplementary file 6 — Figure S6: Raw fluorescent images of western blots showing protein bands detected by NIR700 (near infrared 700 nm) signal (green) scanned on the Odyssey CLX instrument (LiCOR). (A) OVTOKO (lanes 1–4 replicate 1; lanes 5–8 replicate 2; lanes 9–12 replicate 3), (B) OVMANA (lanes 1–4 replicate 1; lanes 5–8 replicate 2), (C) TOV‐21G (lanes 1–4 replicate 1; lanes 5–8 replicate 2) and RMG‐1 (lanes 9–12 replicate 1), (D) RMG‐1 (lanes 1–4 replicate 2; lanes 5–8 replicate 3; lanes 9–12 replicate 4) and (E) OVMANA (lanes 1–4 replicate 3) and TOV‐21G (lanes 5–8 replicate 3). For all blots, vehicle control samples were loaded into lanes 1, 5 and 9; 5 μM ibrutinib treated samples into lanes 2, 6 and 10; 10 μM ibrutinib treated samples into lanes 3, 7 and 11; and 20 μM ibrutinib treated samples into lanes 4, 8 and 12. (F) Licor Chameleon NIR marker showing fluorescent proteins of indicated size (red, green or yellow) with a blue dotted line indicating where nitrocellulose membranes were cut in order to probe with specific antibodies. In panels (A‐E), the upper panels show 3 membrane pieces probed with p‐mTOR (~289 kDa), p‐AKT (~60 kDa) and GAPDH (~37 kDa), and the lower panels show 3 membrane pieces probed with mTOR (~289 kDa), AKT (~60 kDa) and GAPDH (~37 kDa). ^ Indicates a high brightness setting for low abundant proteins. ^^ Indicates a low brightness setting for high abundant proteins. [file CAM4-15-e71795-s006.pdf]
